# Prevalence of Voluntary Counseling and Testing Utilization and Its Associated Factors among Bahirdar University Students

**DOI:** 10.1155/2014/906107

**Published:** 2014-07-03

**Authors:** Getachew Fikadie, Melkamu Bedimo, Zelalem Alamrew

**Affiliations:** ^1^Department of Public Health, College of Medicine and Health Science, Semera University, P.O. Box 132, Samara, Ethiopia; ^2^Department of Public Health, College of Medicine and Health Science, Bahirdar University, P.O. Box 79, Bahirdar, Ethiopia

## Abstract

*Background.* In Ethiopia university students are among the most sexually active and high HIV risk young population group but unfortunately VCT uptake was low (35%–38%) among this group. Examining the factors contributing to VCT uptake is vital to facilitate HIV prevention and control efforts. *Objective.* To assess the prevalence of voluntary counseling and testing utilization and its associated factors among Bahirdar University students in April 2012. *Methods.* Cross-sectional study was conducted in April 2012, among Bahirdar University students. A multistage sampling procedure was used to select 801 students. Data were collected using pretested self-administered questionnaire and analyzed by using SPSS version-16. *Results.* 772 students (79.7% males) participated in the study. The mean age of the respondents' was 21.3. From all respondents 37.8% of the study participants had undergone HIV test. Different variables showed significant association with VCT uptake. *Conclusion.* The major factors identified for increased VCT service utilization were having a friend who got VCT, having discussion about HIV/AIDS with family, origin of residence, year of study, and having boy- or girlfriend. Therefore, actions targeting these predictors are necessary to effectively enhance the use of the VCT services utilization among students.

## 1. Introduction

At the end of 2009 globally it was estimated that there were 33.3 million people living with HIV/AIDS (PLWHA) and there were an estimated 2.6 million people who became newly infected and 1.8 million annual AIDS-related deaths worldwide. Sub-Saharan Africa bears an inordinate share of the global HIV burden. In 2009, there were 22.5 million PLWHA and 1.3 million AIDS related deaths which account for 68% and 72.2% of the global total, respectively [1]. In Ethiopia, PLWHA was estimated at approximately 1.1 million and the prevalence rate was estimated around 2.3% in 2009 [2].

Young people aged 15–24 years are at the forefront of the HIV/AIDS epidemic [3]. They are vulnerable to HIV because of the strong influence of peer pressure and the development of their sexual and social identities. They are also at risk of HIV infection due to their engagement in unsafe sex, injection drug use, exposure to contaminated blood and blood products, or unsterilized skin-piercing procedures [4, 5]. Due to these and related reasons they accounted for 41% of all new HIV infections among adults in 2009 globally. From around 5 million young PLWHA in the world, an estimated 3.9 million young population or 76% were in Sub-Sahara [6]. This age group is the most productive segment of the population that forms the basic education sector which is vital to the creation of human capital [7]. In Ethiopia, youths especially high school and university students are among the populations most vulnerable to HIV infection and HIV/AIDS [8].

This burden necessitates the implementation of different prevention activities and various strategies have been employed to curb the spread of this infection as there is presently no cure [9]. From these strategies, voluntary counseling and testing (VCT) has been introduced in many low resource settings as it helps to create awareness of an individual's HIV status [10]. VCT is the process by which an individual undergoes counseling enabling him or her to make an informed choice about being tested for HIV [11]. With the expansion of antiretroviral therapy, the role of VCT as a gateway for treatment and as a prevention strategy has become an integral part of the global response to HIV/AIDS epidemic [5]. Studies have found that VCT for HIV/AIDS can be an effective intervention in the fight against the disease [12].

Despite the high levels of benefits of voluntary counseling and testing service, VCT utilization was low [12]. Globally only about 40% of people living with HIV know their HIV status. Only 11% of adults in 45 countries of Sub-Saharan Africa received HIV testing in 2009 [7]. The uptake was still low among young and active segment of populations too. Research done in Uganda, Zambia, and among students of Kilimanjaro region revealed that voluntary counseling and testing service utilization was 10%, 14%, and 34.5%, respectively [13–15].

Different research findings in Ethiopia revealed that utilization of voluntary counseling and testing service is low and its level of utilization varies among different segments of the population [16–18]. EDHS (2011) shows that the adult population counseled and tested for HIV was 37% and 38.2% for young people aged 15–24 years [19]. Researches conducted among university and college students also support this fact. For instance, a study in Addis Ababa shows the testing rate was 38.6% among university students [20] and in another study in Debre Birhan it was 35.2% among college students [21] in 2008.

The aim of this study was to assess VCT service utilization and associated factors among Bahir Dar University students, North West Ethiopia, in 2012. The findings of the study will be helpful to expand and improve the service of VCT, contributing to the HIV/AIDS prevention and control programs in the university.

## 2. Methods and Subjects

### 2.1. Study Design and Period

Institution based cross-sectional study has been conducted in April 2012 to assess the prevalence of voluntary counseling and testing utilization and its associated factors among university students.

### 2.2. Study Area

The study has been conducted in Bahirdar University. Bahirdar University is one of the government universities in Ethiopia, which is found in North West part of the country. It is 565 km far from Addis Ababa. The university has four campuses and there were over 15,160 regular undergraduate students during the study period and depending on the discipline it gives for three to six years for first degree programs.

### 2.3. Source Population

The source population for the study was all regular undergraduate Bahirdar University students.

### 2.4. Study Population

Those regular undergraduate students from Bahirdar University in randomly selected department classes of all years of studies (from first year to fifth year (no sixth years during the study period)) were the study population.

### 2.5. Inclusion and Exclusion Criteria

All Bahirdar University undergraduate regular students were illegible to be included in this study. Students attending in nonregular or above first degree programs were not included in the study since they are different from the regular and undergraduate ones with respect to their age, maturity, and employment status.

### 2.6. Sample Size Determination

The sample size was determined by using the formula for a single population proportion. The assumptions made for the sample size calculation were 95% confidence interval (two sided), 5% margin of error, and 38.6% expected proportion VCT uptake among youth [20]. Some 10% was also added to compensate for nonresponse rate and other contingencies. It was multiplied by two for the design effect. A total of 801 university students were needed for the study:

(1) $$\begin{array}{c} n=\frac{{\left(Z_{\alpha /2}\right)}^{2}\times P\left(1-P\right)}{{\left(d\right)}^{2}}+10\%\;\;\text{non}\;\text{response}\;\text{rate}, \end{array}$$

where *n* is the required minimum sample size, *P* is 0.386 (38.6%), *Z*_*α*/2_ is 1.96, *d* is 5% (0.05). *n* = (1.96)^2^(0.386 × 0.614)/(0.05)^2^ = 364, design effect = 2; then 2(364) = 728.

Then total sample size was 728 + 10% = 801.

### 2.7. Sampling Procedure (See Figure 1)

The study participants were selected from target population through multistage sampling technique. A multistage sampling technique was preferred because it is difficult to manage the total 15,160 regular undergraduate students of the university. The sample was proportionally allocated based on the number of the students under the different colleges in the university. From these, two departments have been selected from each by lottery method. There were different years of studies in the selected departments (i.e., first year, second year, third year, fourth year, and fifth year). Again the sample has been allocated proportionally to these years of studies under the selected departments. One class from each year of studies has been selected by lottery method again and the study subjects have been selected from the selected classes by simple random sampling method. The selected students have been provided self-administered questionnaire that would be filled out.

### 2.8. Data Collection Instrument and Management

The data were collected using self-administrated questionnaire. The questionnaire was prepared first in English and then has been translated into Amharic and then translated back to English for the sake of consistency. Before conducting the actual study, the Amharic version questionnaire was pretested at a private college called KEA-Med Medical College among 39 students who completed and returned the questionnaire. The pretest was used to revise its clarity, order of question, skip patterns, and its consistency. Based on the pretest feedback, some questions were rephrased and the final questionnaire was prepared. Four postgraduate students were used as data collection facilitator and to check the completeness of the questionnaire and incompletely filled questionnaires were returned to the respondents so that they fill it in full at the data collection site. The principal investigator also checked the collected data for completeness, accuracy, clarity, and consistency throughout the data collection period.

### 2.9. Data Entry and Analysis

The data entry and analysis have been done by using SPSS version 16 software. Multivariate logistic regression was used to identify significant predictors of VCT service utilization and a *P* value of less than or equal to 0.05 was considered statistically significant.

The bivariate analysis was used to get a preliminary insight into association of each independent variables and dependent variable. However, this simple crude odds ratio and *P* value result may not show the independent variables exact influence on the dependent variable, because the influences of other variables were not controlled. Furthermore, to assess the net effect of each predictor variables on dependent variable multivariate logistic regression was carried out by controlling for the effects of all other intervening variables. To do this, variables with *P* value less than 0.2 (20%) in the bivariate logistic regression were selected and included in multivariate logistic regression to not miss some significant variables.

### 2.10. Ethical Consideration

Ethical clearance was obtained from the institutional research ethics review committee of Bahirdar University. Informed consent was obtained from respondents before they filled questionnaires. They were also assured that the information provided would be used only for research purpose and would therefore be strictly anonymous and dealt with confidentially.

## 3. Results

### 3.1. Demographic Characteristics

From a total of 801 study participants required for the study, 772 self-administered questionnaires were distributed for 772 undergraduate students. Sixteen students did not complete questionnaire and were excluded from analysis and the remaining required thirteen study subjects were unavailable at the time of the survey after three repeated visits resulting in a response rate of 96.4 percent.

As shown in Table 1, majority of the respondents were males and most respondents were in the age range of 20–24 years with the mean age of 21.3 (±SD 1.7). The distribution of the respondents by ethnicity indicated that 79.1 percent of them were from Amhara. The study further revealed that 59.5 percent of responders lived in urban setup before joining BDU. Concerning religious affiliation, most of the respondents were Christians.

**Figure 2:**
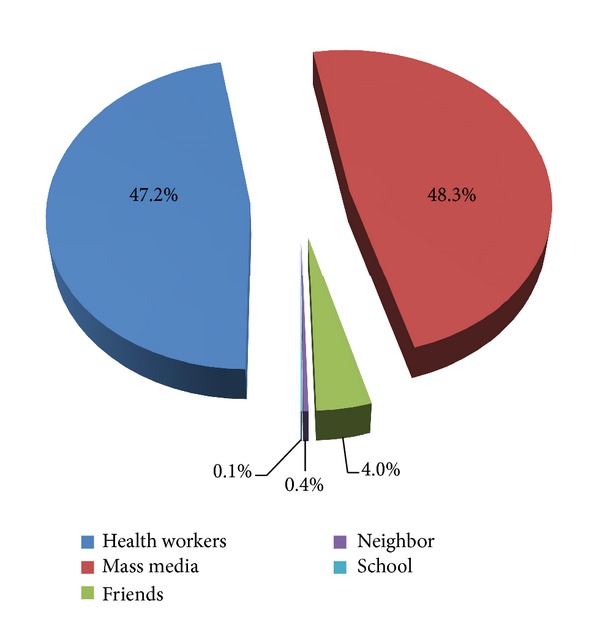
Sources of information for VCT, BDU, April 2012.

All of the respondents have heard of VCT. The most common sources of information were mass media and health institutions (Figure 2). Regarding perceived benefits of VCT, 748 (96.6%) of responders stated that undertaking VCT is important; from these 554 (71.8%) of them said it is important to prevent others, 711 (92.1%) said to take self-care, 601 (77.8%) said to plan for future life, and 502 (65%) to get married (data not shown).

From all respondents 71.8% of them want to have VCT in the future weather they have it before or not, 27.3% of them did not want to have VCT in the future, and the remaining 0.9% did not decide until the data collection time.

From those who did not want to have VCT in the future, fear of friends (35%), fear of confidentiality (25%), no appropriate service time (18%), long waiting service time (8%), self-confident as if negative (12%), and fear to know self (2%) were the reasons they mentioned to not utilize VCT in the future (data not shown).

About three-fourths of the respondents had comprehensive knowledge about HIV/AIDS. Furthermore, 40% of the respondents consider themselves as being at risk of HIV/AIDS infection. Majority (67.8%) of respondents knew someone who was infected with HIV or one who had died of AIDS, 40.5% of them used to discuss with parents HIV/AIDS matters, and 44.2% of them had stigmatized/discriminated attitude (data not shown).

### 3.2. VCT Utilization Practice

The proportion of study participants who ever tested for HIV was 292 (37.8%); from this 77.7% of them were males. Amongst those who had been tested 85 (29.1%), 119 (40.8%), and 85 (29.1%) of them were tested before, 1/2 year, 1 year, and 2 years prior to the data collection, respectively, and one percent of them had been tested during unknown duration (data not shown).

**Figure 3:**
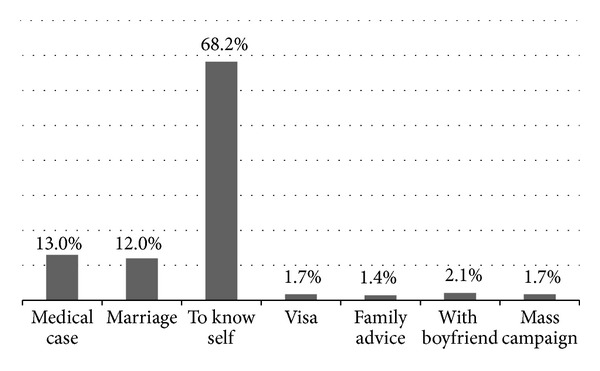
Reasons why they got VCT service before, BDU, April 2012.

Among common reasons mentioned why respondents got VCT service before, as shown from Figure 3, the majority of them were tested to know self or self-check.

**Figure 4:**
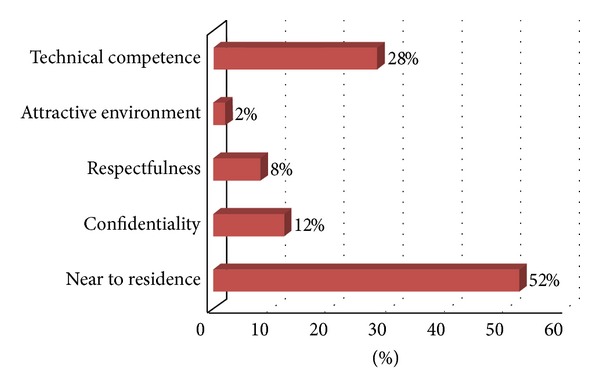
Reason of preference for VCT providing sites BDU, April 2012.

From those who had been tested, seventy-four percent of respondents had been tested from government institutes while twenty-six percent of them were in private institutes. As shown from Figure 4, majority of these respondents prefer the VCT providing sites because the sites were near their residence.

### 3.3. Factors Associated with VCT Uptake

Among the variables included for multivariate logistic regression based on their *P* value in the bivariate analysis, as shown from Table 2, respondents year of study, origin of place, having boy- or girlfriend, knowing someone who was infected with HIV or one who had died of AIDS, having a friend who got VCT testing, and having discussion with parents about HIV/AIDS matters had come out to be important predictors of the likelihood of HIV testing among the study participants.

As indicated above (Table 2), respondents having boy- or girlfriend were 1.6 times more likely to have undergone HIV testing than those who have not (AOR (95% CI) = 1.6 (1.12,2.26)). Individuals from second year were 2.71 times (AOR (95% CI) = 2.71 (1.74,4.22)), from fourth year were 3.54 times (AOR (95% CI) = 3.54 (1.56,8.06)), and from fifth year were 5.87 times (AOR (95% CI) = 5.87 (2.14,16.07)) more likely to have undergone HIV testing than first year counterparts. Those from third year have higher probability of testing, but this increase was not accepted at the minimum *P* value level for significant 0.05.

Those who were from urban area were 1.63 times more likely to be tested as compared to those from rural origin (AOR (95% CI) = 1.63 (1.16,2.30)). Furthermore, respondents who knew someone who was infected with HIV or one who had died of AIDS were 2.2 times more likely to have undergone HIV testing than those who did not (AOR (95% CI) = 2.2 (1.44,3.22)).

Responders who had a friend(s) who had got HIV testing were 2.8 times more likely to be tested than those who had not (AOR (95% CI) = 2.8 (1.99,4.11)). Moreover, responders who had open discussion about HIV matters with their families were 1.8 times more likely to have undergone HIV testing compared to those who had not (AOR (95% CI) = 2.8 (1.26,2.66)).

## 4. Discussion

VCT is considered critical in the fight against HIV/AIDS. Some studies have shown that knowing one's serostatus helps to prevent and control the spread of HIV/AIDS infection [22, 23]. From this study all students have heard about VCT. However, the proportion of study participants who were ever tested for HIV was 37.8%. Similar findings were observed from a study done in Addis Ababa (38.6%) among university students [20] and another study in Debre Birhan (35.2%) among college students [21] in 2008. This finding is higher than in-school youth HIV testing prevalence of Ethiopian BSS II report (9.3%) [24] and another result reported by Abebe and Mitikie in 2009 which was 19% [25]. There might be students from higher institutes who seem to be generally aware of the existence of HIV/AIDS basic facts. Results of this study show that respondent's comprehensive knowledge on HIV/AIDS transmission and misconception was generally high. Out of 772 respondents, 76.2% of them had good comprehensive knowledge of HIV/AIDS. This finding is much higher than Ethiopian BBS II comprehensive knowledge result of all youth's (35.8%) [24]. On the other hand, higher figure was reported from a study among medical students in Nigeria (50.7%) [26] and this might be due to the health related educational background of the study respondents.

By controlling for the effect of other variables through multiple logistic regression, it was found that students with the following characteristics were more likely to have undergone VCT. Those whose origin is from urban area, those who were from second year and above years of studies, those who discussed HIV/AIDS within the family, those who were currently not married but living with sexual partner, those who knew someone who was infected with HIV or one who had died of AIDS, and those who had a friend who got VCT show significant association.

Respondents with second year and above years of studies were more likely to utilize VCT than those who were in the first year. This may be due to the higher possibility that respondents with second or above years of studies could have more access to VCT information when they stayed in the university as well as increased sexual exposure and risk perception. From this study risk perception for HIV/AIDS among responders was increased as their years of study increases 33% in first year, 40% in second year, and 64% in fifth year students. This finding is in agreement with a study done by Madebwe et al. among midlands state university students, Zimbabwe (2012) [27], and another study done by Shingisai among Nelson Mandela Metropolitan university students, South Africa (2011) [28]. On the other hand, a study done in the Tigray, 2008 (among university students), shows year of attainment has no significant association with VCT uptake [29] and this might be due to small sample size used in that study area.

Similarly the likelihood of HIV testing was significantly associated with origin of respondents. Being from urban area had a positive effect on the likelihood of HIV testing. Respondents from urban area were 1.2 times more likely to be tested as compared to respondents from rural area. This finding could be attributed to the opportunity available to the respondents in the urban areas especially the access to information through the print and electronic-media and VCT services tend to be more available in urban areas [30]. The finding of this study is also in line with different studies, like a study done by Hutchinson and Mahlalela in the Eastern Cape, South Africa, 2006 [31], Andarge in Addis Abeba, Ethiopia, 2008 [20], and Dirar in Harar, Ethiopia, 2010 [32].

Discussion with anyone in the family also showed statistically significant and positive association with VCT uptake (AOR (1.83), CI (1.3,2.7)). Open communication in the family might reduce their fear of positive test result and fear of stigma and discrimination. From this study 457 (59%) of responders have discussion with parents about HIV/AIDS matters; of these 273 (60%) of them had nonstigmatized attitude about HIV positive persons. Thus, this might possibly increase their self-efficacy to have VCT compared to nondiscussants. This finding is consistent with most studies done on VCT uptake. A study done by Dirar among youth in the colleges of Harar, Ethiopia (AOR = 1.83) [32], and a national household-based survey of HIV/AIDS that utilized the second-generation surveillance survey approach conducted during 2002 in South Africa (AOR = 1.92) both reported that those who discussed HIV/AIDS prevention with their partner were more likely to have undergone VCT [33]. In contrary to this, those who discussed HIV/AIDS with their family might be because of their prior VCT uptake.

This study also revealed that those who knew someone who was infected with HIV or one who had died of AIDS were 2.15 times more likely to have undergone VCT as compared with their counterparts. This might have increased their knowledge about the burden of HIV/AIDS and advantage of early detection of the disease compared to those who did not know HIV-infected person. This finding is consistent with a study done in South Africa which concludes that knowing someone with HIV increased the likelihood of being tested by about four percent [31]. Another study by Dirar among Harar College students had also reported the same finding [32].

Among the sociodemographic factors, respondents who had boy- or girlfriend were 1.59 times more likely to undertake VCT compared to those who had not. Those who had boy- or girlfriend could have more chance to have VCT as a prerequisite to start sex or they might discuss with their spouse VCT and that might have increased their self-efficacy to have VCT. On the other hand, among those who had no boy/girlfriend, they might think they are not at risk because they might not have had sex yet. Though we cannot state responders sexual activity by using this data, those who had no boy/girlfriend could be involved in high-risk sexual practice in the past and might be afraid of HIV positive test result as a result they might have fear of having VCT. A study in Kenya shows that being involved with someone has a significantly higher probability of using VCT services than not being involved [30]. This finding is also in agreement with a study done in Harar [32] and Mekele [29], Ethiopia. On the other hand, a study done in Guraghe zone (among school youth) [34] reports contradicting result of this finding and this might be due to study population difference (school youth) and the sampling method used (volunteer sampling) where situation is mostly singleness.

This study also shows respondents who had a friend who got VCT service were more likely to undergo VCT than those who had not. Different studies show peers are reported to give support to each other to go for VCT services. A study by Stephen and Florence in Uganda (2009) [35] shows positive peer pressure has a significant effect on utilization of VCT. This report is also in agreement with study by Yahaya et al. (2010) [36] in Nigeria and reported peers are the primary information source for identifying HIV testing services and shows most tested youths had a friend who got VCT. This might show us peers are a key source of information about HIV testing and highlights the potential to use peer networks to promote youth VCT services.

From all respondents who do have high perceived risk, majority (56%) of them did not get tested for HIV/AIDS. This might be because of fear of the possible positive result as they were engaged in unprotected sexual intercourse. A similar study amongst youth in Arbaminch, Ethiopia, has identified fear of positive result affects VCT uptake [37].

## 5. Limitation of the Study

- Findings from this study may not be generalized to the whole population of the young people because the study involved only those young people who are in higher institution.
- As any cross-sectional study, cause and effect relationship was not possible to establish for the factors dealt in the study: for example, VCT uptake with discussion about HIV/AIDS with family.
- The results depended on the responses of the participants and there is a high chance of recall bias.

## 6. Conclusion

All of the respondents have heard about VCT and the proportions of students who have got the test were 37.8%. According to this study the following factors were associated with increased uptake of VCT: those whose origin is from urban area, those who were from second year and above years of study, those who discussed HIV/AIDS within the family, those who do have boy- or girlfriend, those who knew someone who was infected with HIV or one who had died of AIDS, and those who had a friend who got VCT show significant association with VCT utilization.

## 7. Recommendation

Based on the findings of this study, the following recommendations are forwarded.

- VCT counselors are needed to counsel students who come for VCT to inform and initiate their friends. Individuals who got tested should be encouraged to broadcast more information about VCT to university students and friends.
- Because having boy- or girlfriend has positive association with being tested for HIV/AIDS, this study recommends instead of making no attempt to prevent the youth from engagement with someone by instilling fear in them (e.g., a fear of catching HIV/AIDS), we need to openly acknowledge their right to help them pursue it in a more responsible manner.
- Because to be tested for HIV/AIDS is associated with students year of study (being senior) and students origin where they come from (originated from urban), this study recommends interventions implemented should not be in mass but group specific with emphasizing more on year of study (for fresh or new comers) and origin (for rural originated).
- This study recommends student and parent discussion about issues related to HIV/AIDS to increase VCT uptake.
- Students are not going for HIV testing because of high risk perception. On the other hand, knowing someone who was infected with HIV or one who had died of AIDS gives ability to break the bond of terror and increases VCT uptake. This study therefore recommends that individuals who do have HIV virus within their blood better tell their parents about their serostatus and others. At the same time individuals who know someone who died of AIDS better talk about the cause of his/her death with others.

## Figures and Tables

**Figure 1:**
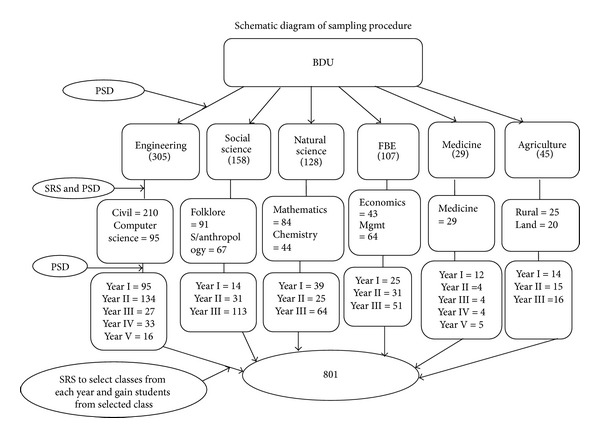


**Table 1:** Demographic characteristics of the university students, BDU, April 2012.

Characteristics	Frequency (*n* = 772)	Percent (%)
Sex		
Male	615	79.7
Female	157	20.3
Age		
15–19	60	7.8
20–24	695	90
≥25	17	2.2
Religion		
Christian	727	94.2
Muslim	45	5.8
Marital status		
Not married	712	92.2
Married	60	7.8
Having boy/girlfriend		
Yes	245	31.7
No	527	68.3
Ethnicity		
Amhara	611	79.1
Oromo	85	11.0
Tigre	29	3.8
Others	47	7
College		
Engineering	305	39.5
Natural science	128	16.6
Medicine and H/sci.	29	3.8
Agriculture	45	5.8
Business and eco.	107	13.9
Social science	158	20.5
Year of study		
First year	199	25.8
Second year	240	31.1
Third year	275	35.6
Fourth year	37	4.8
Fifth year	21	2.7
Place of origin		
Urban	459	59.5
Rural	313	40.5

**Table 2:** Factors associated with VCT uptake, BDU, April 2012.

Variables	VCT utilization		Odds ratio and *P* value			
	Yes	No	COR (95% CI)	*P* value	AOR (95% CI)	*P* value
Marriage						
Not married	262	450	1	0.045	1	0.219
Married	30	30	1.72 (1.01, 2.91)		1.50 (0.79, 2.86)	
Having boy/girlfriend						
No	176	351	1	<0.001	1	**0.01**
Yes	116	129	1.79 (1.32, 2.44)		1.59 (1.12, 2.26)	
Origin of residence						
Rural	110	203	1	0.20	1	**0.005**
Urban	182	277	1.21 (0.90, 1.63)		1.63 (1.16, 2.30)	
Ethnicity						
Amhara	225	386	1	0.036	1	0.214
Oromo	40	45	1.52 (0.97, 2.41)		1.31 (0.79, 2.2)	
Tigrie	15	14	1.84 (0.87, 3.88)		1.74 (0.77, 4.0)	
Others	12	35	0.59 (0.30, 1.16)		0.65 (0.31, 1.36)	
Faculty						
Engineering	121	184	1	0.081	1	0.296
Natural science	45	83	0.82 (0.54, 1.27)		0.98 (0.57, 1.67)	
Medicine	3	26	0.18 (0.05, 0.59)		0.25 (0.06, 1.04)	
Agriculture	21	24	1.3 (0.71, 2.5)		0.93 (0.44, 1.96)	
FBE	40	67	0.91 (0.58, 1.43)		1.30 (0.71, 2.35)	
Social science	62	96	0.98 (0.66, 1.46)		1.33 (0.79, 2.24)	
Year of study						
First year	50	149	1	<0.001	1	**<0.001**
Second year	116	124	2.79 (1.85, 4.19)		2.71 (1.74, 4.22)	
Third year	97	178	1.62 (1.08, 2.43)		1.35 (0.87, 2.10)	
Fourth year	17	20	2.53 (1.23, 5.21)		3.54 (1.56, 8.06)	
Fifth year	12	9	3.97 (1.58, 9.99)		5.87 (2.14, 16.07)	
Perceived at risk						
No	141	284	1	0.009	**1**	0.117
Yes	137	172	1.60 (1.19, 2.2)		1.43 (1.02, 2.0)	
Do not know	14	24	1.18 (0.6, 2.3)		1.16 (0.54, 2.5)	
Perceived benefit						
No	6	18	1	0.19	1	0.202
Yes	286	462	1.86 (0.73, 4.73)		1.92 (0.71, 5.2)	
Knew someone who was infected/died with HIV						
No	48	200	1	<0.001	1	**<0.001**
Yes	244	280	3.63 (2.53, 5.2)		2.15 (1.44, 3.22)	
Having a friend who got VCT						
No	61	238	1	<0.001	1	**<0.001**
Yes	231	242	3.72 (2.7, 5.2)		2.79 (1.99, 4.11)	
Discussed with parents HIV						
No	80	235	1	<0.001	1	**0.001**
Yes	212	245	2.54 (1.86, 3.45)		1.83 (1.26, 2.66)	
Stigmatized attitude						
Yes	112	229	1	0.011	1	0.269
No	180	251	1.47 (1.09, 197)		1.21 (0.86, 1.70)	

## Acronyms

BDU: Bahirdar University
PLWHA: People living with HIV/AIDS
VCT: Voluntary counseling and testing
EDHS: Ethiopia Demographic and Health Survey.

## References

[1] UNAIDS/WHO (2010). *Report on the Global HIV/AIDS Epidemic*.

[2] Federal Democratic Republic of Ethiopia (2010). Report on progress towards implementation of the UN declaration of commitment on HIV/AIDS.

[3] UNAIDS (2004). *Report on the Global AIDS Epidemic 2004: Executive Summary*.

[4] UNAIDS/WHO (2006). *Report on the Global HIV/AIDS Epidemic*.

[5] UNAIDS/WHO (2004). *Report on the Global HIV/AIDS Epidemic*.

[6] UNAIDS (2011). *Synthesis of Strategic Information on HIV and Young People. Securing the Future Today*.

[7] WHO/UNAIDS/UNICEF (2009). *Report on the Global HIV/AIDS Epidemic*.

[8] USAIDS (September 2010). *HIV/AIDS Profile Report: Ethiopia*.

[9] UNAIDS Tools for evaluating HIV voluntary counseling and testing: best experience collection.

[10] Menzies N, Abang B, Wanyenze R (2009). The costs and effectiveness of four HIV counseling and testing strategies in Uganda. *AIDS*.

[11] UNAIDS (1999). *Policy Statement on HIV Testing and Counseling*.

[12] UNAIDS (August 2001). *The Impact of Voluntary Counseling and Testing: A Global Review of the Benefits and Challenges*.

[13] Mugisha E, Van Rensburg GH, Potgieter E (2011). Strategic framework for increasing accessibility and utilization of voluntary counseling and testing services in uganda. *AIDS Research and Treatment*.

[14] Ministry of Health (2009). HIV testing and counseling in clinical settings. *Annual Report*.

[15] Ministry of Health and Child Welfare (2008). *HIV/AIDS: A Comprehensive Approach*.

[16] Federal Democratic Republic of Ethiopia Federal HIV/AIDS Prevention and Control Office (March 2010). *Report on Progress towards Implementation of the UN Declaration of Commitment on HIV/AIDS*.

[17] Wondwossen M (2007). *Assessment of VCT utilization for HIV/AIDS among government and non-government employees in Butajira, SNNPR, Ethiopia [MPH thesis]*.

[18] Admassu M, Fitaw Y (2006). Factors affecting acceptance of VCT among different professional and community groups in North and South Gondar Administrative zones, North West Ethiopia. *Ethiopian Journal of Health Development*.

[19] CSA [Ethiopia] and ICF International Ethiopia Demographic and Health Survey 2011.

[20] Andarge A (2008). *Determinants of voluntary HIV counseling and testing among Addis Ababa university undergraduate final year students [Ph.D. thesis]*.

[21] Gashaw Z (2008). Knowledge, attitude towards practicing of voluntary HIV counselling testing and the determinants of VCT uptake: a case study in Debre Birhan Teachers Training College. *Ethiopian Journal of Health Development*.

[22] Bunnell R, Ekwaru JP, Solberg P (2006). Changes in sexual behavior and risk of HIV transmission after antiretroviral therapy and prevention interventions in rural Uganda. *AIDS*.

[23] Arthur G, Nduba V, Forsythe S, Mutemi R, Odhiambo J, Gilks C (2007). Behaviour change in clients of health centre-based voluntary HIV counselling and testing services in Kenya. *Sexually Transmitted Infections*.

[24] MOH/HAPCO (2005). *HIV/AIDS Behavioral Surveillance Survey (BSS) Ethiopia 2005 Round Two*.

[25] Abebe A, Mitikie G (2009). Perception of high school students towards voluntary HIV counseling and testing, using health belief model in Butajira, SNNPR. *Ethiopian Journal of Health Development*.

[26] Daniyam CA, Agaba PA, Agaba EI (2010). Acceptability of voluntary counselling and testing among medical students in Jos, Nigeria. *Journal of Infection in Developing Countries*.

[27] Madebwe V, Crescentia M, Lilian P, Kudakwashe C (2012). Taking the test: voluntary counselling and testing (VCT) among midlands state university students. *Education Research Journal*.

[28] Shingisai M (2011). *Factors Influencing University Students’ Use of HIV Voluntary Counselling and Testing Services: An Analysis Using the Health Belief Model*.

[29] Bayray A (2010). Knowledge, attitude, and practice of voluntary counseling and testing for HIV among university students, Tigray, Northern Ethiopia. *MUJS*.

[30] Julie A Determinants of using voluntary counseling and testing for HIV/AIDS in Kenya.

[31] Hutchinson PL, Mahlalela X (2006). Utilization of voluntary counseling and testing services in the Eastern Cape, South Africa. *AIDS Care*.

[32] Dirar A (2010). Assessment of factors contributing to voluntary counseling and testing uptake among youth in the Colleges of Harar, Ethiopia. *Harar Bulletin of Health Sciences*.

[33] Simbayi O, Chauveau J, Ramlagan S (2003). Determinants of the use of voluntary counselling and testing services among the sexually active adult population of South Africa. *Antiviral Therapy*.

[34] Wondimagegn G (2004). *Factors associated with VCT utilization in Guraghe zone, SNNPR, Ethiopia [MPH. thesis]*.

[35] Stephen S, Florence N (2009). Voluntary counseling and testing services: breaking resistance to access and utilization among the youths in Rakai district of Uganda. *Educational Research and Reviews*.

[36] Yahaya L, Jimoh G, Balogun O (2010). Factors hindering acceptance of HIV/AIDS Voluntary Counseling and Testing (VCT) among youths in Kwara State, Nigeria. *Nigeria. Journal of AIDS and HIV Research*.

[37] Meshesha B (2007). *Factors influencing voluntary counseling and testing service utilization associated with VCT utilization among youths in Arbaminch town: a health belief model approach. Southern Ethiopia [M.S. thesis]*.

